# Development of a behaviour change workplace-based intervention to improve nurses’ eating and physical activity

**DOI:** 10.1186/s40814-021-00789-0

**Published:** 2021-02-18

**Authors:** Brian T. Power, Kirsty Kiezebrink, Julia L. Allan, Marion K. Campbell

**Affiliations:** 1 Department of Health and Nutritional Sciences, Institute of Technology Sligo, F91 YW50, Sligo, Republic of Ireland; 2 Health Services Research Unit, University of Aberdeen, Aberdeen, AB25 2ZD Scotland UK; 3 Nutrition and Dietetics, University College London Hospitals NHS Foundation Trust (UCLH), London, NW1 2BU UK; 4 Health Psychology, Institute of Applied Health Sciences, University of Aberdeen, AB25 2ZD, Aberdeen, Scotland UK

**Keywords:** Programme, Behavioural interventions, Behaviour change, Nurses, Physical activity, Exercise, Diet, Healthcare professionals

## Abstract

**Background:**

There is a critical need for an intervention to improve nurses’ eating and physical activity behaviours. As nurses spend a substantial proportion of their waking hours at work, concerted efforts to deliver such interventions in the workplace is growing. This study formed part of a multiphase programme of research that aimed to systematically develop an evidence-based and theory-informed workplace intervention to promote changes in eating and physical activity among nurses.

**Methods:**

The intervention was developed iteratively, in line with Medical Research Council complex intervention guidelines. It involved four activities: (1) identifying the evidence base, (2) understanding the determinants of nurses’ eating and physical activity behaviour change through theory-based qualitative interviews and survey, (3) identifying intervention options using the Behaviour Change Wheel, and (4) specifying intervention content and implementation options using a taxonomy of behaviour change techniques.

**Results:**

Data from 13 randomised controlled trials indicated that workplace-based behaviour change interventions targeted to this population are effective in changing behaviour. The evidence base was, however, limited in quantity and quality. Nurses’ beliefs about important factors determining their eating and physical activity behaviour were identified across 16 qualitative interviews and 245 survey responses, and key determinants included environmental context and resources, behavioural regulation, emotion, beliefs about consequences, knowledge and optimism. Based on these findings, 22 behaviour change techniques suitable for targeting the identified determinants were identified and combined into a potential workplace intervention.

**Conclusions:**

An evidence-based and theory-informed intervention tailored to the target population and setting has been explicitly conceptualised using a systematic approach. The proposed intervention addresses previous evidence gaps for the user population of nurses. Further to this, such an intervention, if implemented, has the potential to impact nurses’ eating and physical activity behaviours and in turn, the health of nurses and the quality of healthcare delivery.

**Supplementary Information:**

The online version contains supplementary material available at 10.1186/s40814-021-00789-0.

## Background

Nurses, comprising approximately half the health workforce, are crucial to an effective healthcare system. Yet, poor eating behaviours and physical inactivity among nurses have been reported across a range of different countries [1–4]. In the United Kingdom (UK) and the USA, studies have reported that over half of nurses are overweight or obese [2, 5]. Suboptimal eating and physical activity patterns among nurses have many implications. For instance, obesity and concomitant health problems such as musculoskeletal disorders may be an important determinant of sickness absence among nurses [6]. Absenteeism in nurses may create overload among the healthcare staff that remain, reducing workforce capacity with inherent risks to the delivery of quality care to patients [7].

Given global nursing shortages, the retention of a high-quality nursing workforce is a key policy issue for healthcare system sustainability [8]. Policy-makers have therefore emphasised the importance of developing effective and cost-effective interventions to support nurses’ eating and physical activity and subsequent health [9]. As a case in point, the international movement of the Health Promoting Hospitals and Services network stresses the urgent need to improve the health behaviour of nurses [10]. Workplaces are particularly advantageous settings for the delivery of behaviour change interventions as they enable repeated access to a relatively fixed population who can spend up to one third of their waking lives at work [11, 12]. Improved eating and physical activity could therefore be consistently enacted over time, maximising chances of sustained improvement of health outcomes. This is a particularly pertinent advantage given the recognised difficulty in long-term maintenance of behaviour change [13].

To date, a limited number of interventions to improve nurses’ eating and physical activity have been developed [14–16]. Despite evidence of effectiveness, previous intervention development work in this area has not been reported in sufficient detail to allow analysis of specific individual intervention components. Theoretical approaches have also rarely been reported. This is important as interventions based on theory have been identified as most effective at changing health behavior [17]. The lack of empirical evidence on the ‘active ingredients’ of these interventions makes it difficult to draw conclusions about what works within workplace interventions to change nurses’ eating and physical activity behaviours. This reduces the replication potential of interventions, which is a crucial part of cumulative science [18, 19].

The Medical Research Council (MRC) framework for developing and evaluating complex interventions advises that an iterative development process should be undertaken for maximum replication potential and impact [20]. This iterative process should include identification of the evidence base, development of a theoretical understanding of target behaviors and a modelling of processes and outcome. The present study investigated the existing evidence base with a systematic review, identified relevant theoretical determinants of the target behaviours with interviews and a survey informed by the Theoretical Domains Framework (TDF) [21] and modelled the processes of change/identified appropriate methods of changing behaviour using the Behaviour Change Wheel (BCW) [22] and Behaviour Change Technique Taxonomy version 1 (BCTTv1) [23]. As an overarching theoretical framework, the TDF consists of 14 domains from several theories of health behaviour change that can influence eating and physical activity. The BCW is another framework that integrates health behavior change theory and consists of nine intervention functions to describe how an intervention might change behavior and seven policy categories that can be used to support the implementation of the intervention functions. The BCW is supported by BCTTv1 which is a taxonomy of 93 replicable behaviour change techniques (BCTs). Undertaking the process of using the TDF, BCW and BCTTv1 ensures that any developed intervention is tailored to the needs of the target population.

These methods mirror those used for systematically developing interventions across a variety of populations and behaviours. For example, Murphy et al. used the TDF and BCW to develop a capacity-building intervention to promote pharmacists’ roles in mental health care [24]. The added value of this approach is that it supports the selection of appropriate BCTs from the BCTTv1 [23]. BCTs are the ‘active ingredients’ of interventions designed to bring about change in behaviours such as nurses’ eating and physical activity. Recent mapping exercises by Cane et al. [25] and Michie et al. [26] have identified the most appropriate BCTs to use to change each of the possible theoretical determinants of behaviour identified within the TDF domains.

The aim of the present paper is to describe the development of a new tailored and cohesive workplace behavior change intervention which targets nurses’ eating and physical activity behaviours using the TDF, BCW and BCTs. It has been developed to an appropriate stage for evaluation in a feasibility study.

## Methods

### Design

**Fig. 1:**
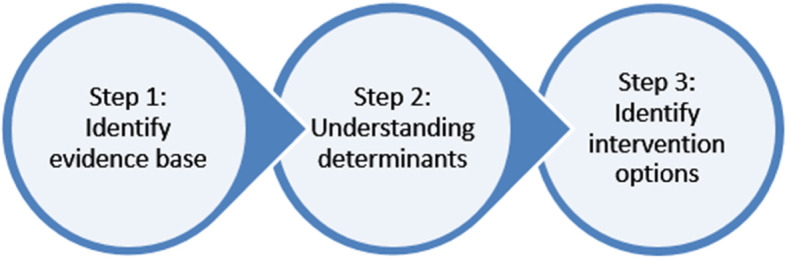
Systematic step-wise intervention development process

The development process is illustrated in Fig. 1. Reporting of the developed intervention is consistent with the recommendations of the Template for Intervention Description and Replication (TIDieR) checklist [27].

Intervention development involved four key steps with the procedure for these steps outlined below.

### Procedure

#### (1) Identifying the evidence base

Step one drew upon the evidence base through a systematic review of the literature. The aims of the systematic review were to (1) investigate the effectiveness of workplace-based dietary and/or physical activity interventions targeting healthcare professional groups such as nurses, (2) identify and describe key components of effective interventions, (3) identify theoretical models of behaviour change involved in effective interventions and (4) investigate whether intervention effectiveness in this setting is improved by the extent to which interventions are explicitly developed based on theory. The methods of this systematic review are reported in full elsewhere [15].

#### (2) Understanding the theoretical determinants of nurses’ eating and physical activity behaviours

This step reflects a behavioral diagnosis, whereby reasons for nurses’ unhealthy eating and physical activity behaviours and what needs to change to improve nurses eating and physical activity behaviors were identified. Semi-structured qualitative interviews followed by a quantitative survey were undertaken based on the TDF. The TDF is an overarching framework that summarises 84 known determinants of behaviour into fourteen theoretical construct domains [21], allowing the systematic investigation of the psychological determinants which may need to be addressed in order to achieve behaviour change [21]. The methods of the qualitative interview component of this step are reported elsewhere [28]. A separate 113-item survey based on the determinants identified in these qualitative interviews was also constructed and completed by 245 nurses (226 females, 17 males; recruited from hospitals based in the UK) [29]. This enabled quantitative estimation of the relative importance of different determinants.

The current paper focusses on step three in more detail. Specifically, how the findings from the behavioural diagnosis above were mapped onto the BCW framework [22] and BCTTv1 [30] to systematically develop a workplace behaviour change intervention for nurses. The current paper therefore builds on the empirical work generated from steps one and two.

#### (3) Identifying intervention options

Having previously identified the theoretical determinants of nurses’ eating and physical activity behaviour change using the TDF in step two [28], with details outlined in the Results section of the current article, the BCW [22, 23] was used to identify how these theoretical determinants of nurses’ eating and physical activity behaviour could be targeted. The BCW is a synthesis of 19 behaviour change frameworks [22] which helps to organise and identify intervention types (‘intervention functions’) and policy strategies (‘policy categories’) that are likely to be effective in changing particular theoretical determinants (TDF domains) of target behaviours such as eating and physical activity [22, 23]. In the present study, published matrices [23] were used to identify the most appropriate intervention and policy options for changing the theoretical determinants of nurses’ eating and activity behaviour identified during the TDF-informed interviews and survey.

Once broadly appropriate intervention and policy categories had been identified, specific BCTs were selected using published matrices and mapping documents developed by Cane et al. [25] and Michie et al. [23] which allowed specific techniques associated with particular theoretical determinants and intervention types to be identified. The evidence for the potential effectiveness of each selected BCT was evaluated by conducting a rapid scoping review. The likely parameters of effectiveness for each technique (i.e. the circumstances under which each technique would be expected to work) were also established by consulting recommendations of the Iterative Protocol for Evidence Base Accumulation [31] and using the intervention mapping taxonomy of BCTs [32].

#### (4) Feasibility screening of developed intervention

This process was guided by the APEASE criteria (Affordability, Practicability, Effectiveness/cost-effectiveness, Acceptability, Side effects/safety, Equity). In applying these criteria, acceptability, practicability and affordability were considered as they are more appropriate for the early phases of intervention development. By contrast, effectiveness, side effects/safety, equity would be more applicable to the intervention evaluation phase following a full-scale trial.

## Results

### The evidence base

The systematic review results identifying the evidence base that laid the foundation for intervention development are reported in detail elsewhere [15] and summarised briefly in this section. Thirteen randomised controlled trials were retrieved. Despite evidence of effectiveness of workplace-based interventions to change nurses’ eating and activity behaviours, the available evidence base was limited in quality and quantity. Several important gaps were noted which caused uncertainty in establishing what intervention content and characteristics contributed most to intervention effectiveness. Additionally, few interventions to change nurses’ eating and physical activity were underpinned by a coherent theoretical framework or formative research.

### Theoretical determinants of nurses’ eating and physical activity behaviour

Details of the qualitative interview study are reported elsewhere [28]. In summary, across the 16 qualitative interviews and 245 survey responses, the three most important barriers to nurses’ eating and physical activity behaviour change related to environmental context and resource factors such as time and the food environment, emotional factors such as mood and stress and behavioural regulation factors such as lack of self-monitoring and planning. The three most important enablers identified were knowledge of relevant guidelines and strategies for changing eating and physical activity behaviour, optimism about likely outcomes of behaviour change attempts and beliefs about the likely positive consequences of healthy eating and physical activity.

### Intervention options

Nine intervention functions and seven policy categories from the BCW were identified as having potential to bring about eating and physical activity behaviour change in nurses (Tables 1 and 2).

**Table 1:** TDF domain and intervention function matrix

TDF Domains	Intervention functions (Definitions)								
	Education (increasing knowledge or understanding)	Persuasion (using communication to induce positive or negative feelings or stimulate action)	Incentivisation (creating expectation of reward)	Coercion (creating expectation of punishment or cost)	Training (imparting skills)	Restriction (using rules to reduce the opportunity to engage in the behaviour (or to increase behaviour by reducing opportunity to engage in competing behaviours)	Environmental restructuring (changing the physical or social context)	Modelling (providing an example for people to aspire to or imitate)	Enablement (increasing means/reducing barriers to increase capability or opportunity)
**Knowledge**	x								
**Optimism**	x	x						x	x
**Beliefs about consequences**	x	x						x	
**Environmental context and resources**					x	x	x		x
**Emotion**		x	x	x				x	x
**Behavioural regulation**	x				x			x	x

*Selection of intervention function indicated with an X

**Table 2:** Intervention function and policy category matrix

Policy categories (Definition)	Intervention functions								
	Education	Persuasion	Incentivisation	Coercion	Training	Restriction	Environmental restructuring	Modelling	Enablement
Communication/ marketing **(using print, electronic, telephonic or broadcast media)**	X	X	X	X				X	
Guidelines **(creating documents that recommend or mandate practice. This includes all changes to service provision)**	X	X	X	X	X	X	X		X
Fiscal measures **(using the tax system to reduce or increase the financial cost)**			X	X	X		X		X
Regulation **(establishing rules or principles of behaviour or practice)**	X	X	X	X	X	X	X		X
Legislation **(making or changing laws)**	X	X	X	X	X	X	X		X
Environmental/social planning **(designing and/or controlling the physical or social environment)**							X		X
Service provision **(delivering a service)**	X	X	X	X	X			X	X

*Selection of policy category indicated with an X

Based on BCTs previously judged by a consensus of four experts in behaviour change to be appropriate for changing each selected intervention function [23], 89 BCT’s likely to be suitable to change the identified determinants were initially considered for selection. Guided by a previous expert rating study [25], 22 out of the possible 89 BCTs were selected as those most likely to be effective in changing the factors identified in the qualitative interviews/ survey as likely determinants of nurses’ eating and physical activity (Table 3). Additionally, it was found that certain combinations of BCTs may interact with each other to amplify or reduce effectiveness. For example, the evidence suggests that *Self-monitoring of behaviour* and subsequent *Feedback* are a typically effective combination of BCTs. However, the BCT: *Threat (future punishment) may include fear arousal* is likely to be counter effective when self-efficacy is low. There were insufficient studies retrieved that examined which specific BCTs or BCT combinations were most effective for distinct target populations and settings.

**Table 3:**
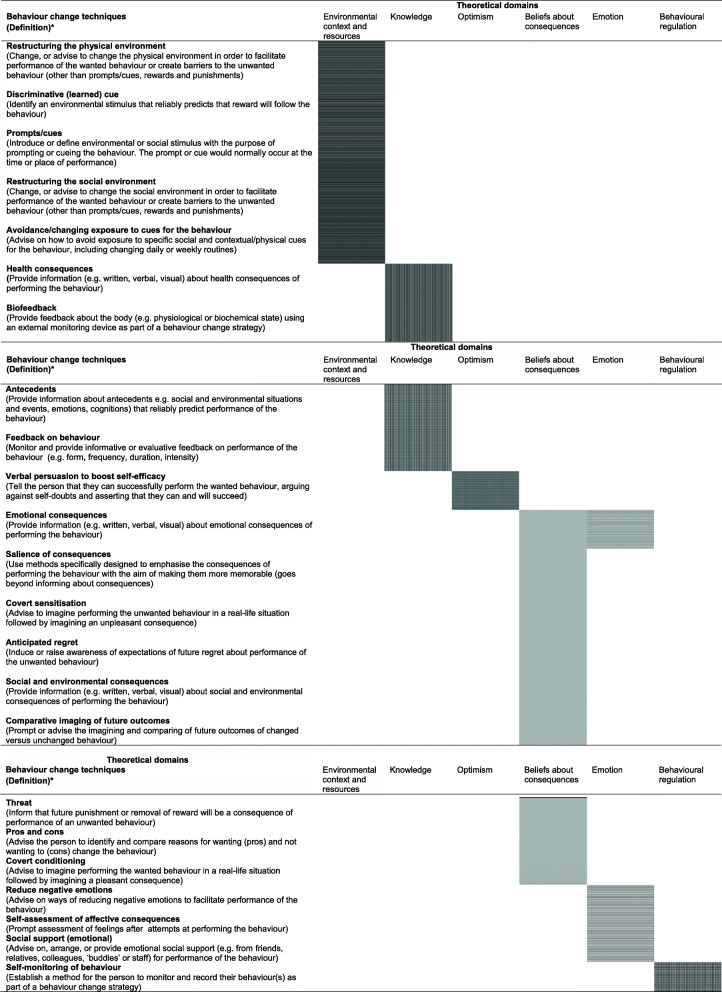
BCT and TDF Matrix

*BCT definitions based on [30].

Further information about the selected BCTs is outlined in detail within Additional file 1.

Table 4 contains a full description of the proposed (or suggested) intervention components. It summarises the theoretical determinants of nurses’ eating and activity behaviours, the BCTs likely to be able to change these determinants and how these techniques may be translated in practice. The intervention is multimodal, involving a combination of digital and printed modes of delivery. A multimodal approach to intervention delivery was adopted as it is more likely to enhance effectiveness and appeal to a wider range of nurse preferences. The completed TIDieR checklist also provides further details about BCT operationalisation examples including the number, duration, intensity and dose of the digital sessions (Additional file 2). A brief summary of the proposed intervention that arose from the systematic step-wise process adopted in the current study is presented below. This scenario represents the result of a creative process using the TDF, BCW and BCTTv1.

**Table 4:** Theoretical Domains Framework target determinants, behaviour change techniques (BCT) and BCT operationalisation within the workplace intervention

Determinant targeted by Behaviour change technique	Behaviour change technique	An example of what the behaviour change technique would look like within the workplace intervention	Mode of delivery
Environmental context and resources	Restructuring the physical environment	Vinyl footsteps placed on hospital floors to promote stair walking Smaller portion sizes provided as substitutes in the workplace canteen	Printed material
	Avoidance/changing exposure to cues for the behaviour	Online-based self-completed session (Module Session 1); • Recommendations to i) Determine appropriate servings in advance when eating foods (either in workplace/out of workplace); ii) Focus on activities other than watching television and going to pubs and bars which might have previously been associated with sedentary behaviour	Digital—individual
	Prompts/cues	Vinyl footsteps placed on hospital floors to promote stair walking Food within workplace calorie labelled at the point of purchase	Printed material
	Restructuring the social environment	Social marketing campaign with branding. For branding, a professionally created logo placed across all intervention components	Digital—individual
	Discriminative (learned) cue	Online-based self-completed session (Module Session 1); • Advises nurses’ about taking the stairs during the working day but not the lift and provide information about entry to a draw for a non-monetary incentive	Digital—individual
Behavioural regulation	Self-monitoring of behaviour	Online-based self-completed session (Module Session 1); • Pedometer for tracking daily footsteps on personal page of online-based programme • Nurses requested to keep a record of unhealthy snacking/physical inactivity on personal page of online-based programme	Digital—individual
Knowledge	Biofeedback	Online-based self-completed session (Module Session 2); • Invite nurses to undertake a fitness and strength test	Digital—individual
	Information about health consequences	A brief message about the increased risk of chronic disease following unhealthy dietary/physical activity patterns placed on pedometer in conjunction with advice on how to mitigate this risk	Printed material
	Antecedents	Online-based self-completed session (Module Session 2); • Nurses requested to keep a record of unhealthy snacking/physical inactivity on personal page of online-based programme. This is to identify situations or events occurring prior to unhealthy snacking/physical inactivity	Digital—individual
	Feedback on behaviour	Online group-based motivational interviewing session (Module Session 3); • A registered dietitian/nutritionist/physiotherapist provides feedback within motivational interviewing session on healthy eating/physical activity progress	Digital—grouped
Optimism	Verbal persuasion to boost self-efficacy	Online group-based motivational interviewing session (Module Session 3); • Nurses verbally persuaded to enhance self-efficacy as part of online group-based motivational interviewing session	Digital—grouped
Beliefs about consequences	Information about emotional consequences	Online-based self-completed session (Module Session 2) Nurses advised to track their mood alongside their eating and physical activity behaviours on personal page of online-based programme. This is to support nurses identify the emotional consequences of healthy and unhealthy behaviours for themselves.	Digital—individual
	Salience of consequences	Online-based self-completed session (Module Session 2) Nurses requested to keep a record of the dangers of eating unhealthy/being sedentary on personal page of online-based programme. Concrete information on what will happen if a nurse does this every day (e.g. if you eat like this every day for the next 4 weeks you will lose/gain X kg) provided to increase the personal salience of the consequences of unhealthy behaviours	Digital—individual
	Anticipated regret	Online group-based motivational interviewing session (Module Session 3) Nurses requested to answer the following two questions (“If I did not eat healthily and participate in physical activity I would later feel regret.”) and (“If I did not eat healthily and participate in physical activity, I would later wish I had.”)	Digital—grouped
	Information about social and environmental consequences	A web-based staff newsletter and social media used for communicating information about social and environmental consequences of healthy eating and physical activity	Digital—individual
	Comparative imaging of future outcomes	Online-based self-completed session (Module Session 2) Prompt nurses to imagine and compare likely or possible outcomes following eating healthily/participating in physical activity versus not performing these behaviours	Digital—individual
Beliefs about consequences	Vicarious reinforcement	Online group-based motivational interviewing session (Module Session 3) A registered dietitian/nutritionist/physiotherapist would provide feedback on healthy eating/physical activity progress	Digital—grouped
	Threat (future punishment)	A fear appeal message in combination with a self-affirmation message incorporated into social marketing campaign	Digital—grouped
	Pros and cons	Online-based self-completed session (Module Session 4) Nurses requested to list and compare the advantages and disadvantages of eating healthy/physical activity participation	Digital—individual
Emotion	Reduce negative emotions	Online-based self-completed session (Module Session 4) Expert video discussing the use of stress management techniques such as progressive muscular relaxation and diaphragmatic breathing to help nurses manage their stress	Digital—individual
	Self-assessment of affective consequences	Online-based self-completed session (Module Session 4) Advises nurses to record how they feel after eating healthily/participating in physical activity on personal page of online-based programme	Digital—individual
	Social support (emotional)	Online-based group-based forum and discussion (Module Session 5) Participants could share experiences and give each other tips or search for training partners	Digital—grouped

Nurses would have access to an online programme with a personal page consisting of five modules. Module 1 contains recommendations on avoidance/changing exposure to cues for eating and physical activity behaviour such as determining appropriate servings in advance when eating (BCT: *Avoidance/changing exposure to cues for the behaviour*). It could also include nurses keeping a record of unhealthy snacking/physical inactivity on their personal page (BCT: *Self-monitoring of behaviour*)*.* Module 2 prompts nurses to imagine and compare likely or possible outcomes following eating healthily/participating in physical activity versus not performing these behaviours (BCT: *Comparative imaging of future outcomes*), invites nurses to undertake a fitness and strength test (BCT: *Biofeedback*) and identify situations or events occurring prior to unhealthy snacking/physical inactivity (BCT: *Antecedents*). Nurses would be advised to track their mood alongside their eating and physical activity behaviours on personal page of online-based programme so as to identify the emotional consequences of healthy and unhealthy behaviours for themselves (BCT: *Information about emotional consequences*). Lastly, module 2 would also incorporate information to increase the personal salience of the consequences of unhealthy eating and physical activity behaviours (BCT: *Salience of consequences*).

Module 3 contains an expert video discussing the use of stress management techniques such as progressive muscular relaxation and diaphragmatic breathing to help nurses manage their stress (BCT: *Reduce negative emotions*). A registered dietitian/nutritionist/physiotherapist would also provide feedback within a motivational interviewing session on healthy eating/physical activity progress (BCT: *Feedback on behaviour*; BCT: *Vicarious reinforcement*) and verbally persuaded to enhance self-efficacy (BCT: *Verbal persuasion to boost self-efficacy*). The motivational interviewing session would also entail nurses providing their level of agreement/disagreement with the following two statements (“If I did not eat healthily and participate in physical activity I would later feel regret”) and (“If I did not eat healthily and participate in physical activity, I would later wish I had”) (BCT: *Anticipated regret*). Further, nurses would be advised to record how they feel after eating healthily/participating in physical activity on the personal page of Module 4 (BCT: *Self-assessment of affective consequences*) undertake a fitness and strength test and record how they feel after eating healthily/participating in physical activity (BCT: *information about emotional consequences*).

Module 5 contains forum and discussion pages for peer to peer social support (BCT: *Social support (emotional)*). In addition, nurses would be invited to attend online group motivational interviewing sessions to boost self-efficacy (BCT: *Verbal persuasion to boost self-efficacy*). Prior to each session, nurses would be requested to self-monitor eating and physical activity behaviours (BCT: *Self-monitoring of behaviour*). Similarly, at a separate online group session, nurses would be requested to describe and compare the advantages and disadvantages of eating healthy/physical activity participation (BCT: *Pros and cons*).

Coupled with digital intervention delivery, the workplace physical environment would also be restructured by the following evidence-based strategies; placing vinyl footsteps on hospital floors to promote stair walking (BCT: *Restructuring the physical environment*), smaller portion sizes would be provided in the hospital canteen and food would be calorie labelled at the point of purchase (BCT: *Prompts/cues*). Nurses could also be advised about entry to a draw for a non-monetary incentive for taking the stairs during the working day but not the lift (BCT: *Discriminative (learned) cue*). A brief message about the increased risk of chronic disease following unhealthy dietary/physical activity patterns could be placed on distributed pedometers in conjunction with advice on how to mitigate this risk (BCT: *Information about health consequences*). To restructure the social environment, social norm messages via a social marketing campaign would be implemented (BCT: *Restructuring the social environment*). Social marketing campaign involving a fear appeal message in combination with a self-affirmation message would also be implemented (BCT: *Threat (future punishment*)). A logo would be professionally created and utilised across all intervention components to promote the intervention. A web-based staff newsletter and social media used for communicating information about social and environmental consequences of healthy eating and physical activity would also be implemented (BCT: *Information about social and environmental consequences*).

### Feasibility screening of developed intervention

Table 5 displays the details of the APEASE evaluation carried out on the candidate intervention components.

**Table 5:** Feasibility screening of developed intervention (guided by a subset of APEASE criteria)

Candidate intervention components	Mode of delivery	Feasibility
Vinyl footsteps placed on hospital floors to promote stair walking. Smaller portion sizes provided as substitutes in the workplace canteen.	Printed material	Affordable—yes Practical—yes Acceptable—yes
Online-based self-completed session (Module Session 1); • Recommendations to i) Determine appropriate servings in advance when eating foods (either in workplace/out of workplace); ii) Focus on activities other than watching television and going to pubs and bars which might have previously been associated with sedentary behaviour	Digital—individual	Affordable—yes, however increase digital functionality may increase cost Practical—yes Acceptable—yes, however, further piloting of online activities would be necessary to confirm
Vinyl footsteps placed on hospital floors to promote stair walking Food within workplace calorie labelled at the point of purchase	Printed material	Affordable—yes Practical—yes Acceptable—yes
Social marketing campaign with branding. For branding, a professionally created logo placed across all intervention components	Digital—individual	Affordable—yes Practical—yes Acceptable—yes with participant involvement in designing messages for the social marketing campaign
Online-based self-completed session (Module Session 1); • Advises nurses about taking the stairs during the working day but not the lift and provide information about entry to a draw for a non-monetary incentive	Digital—individual	Affordable—yes, however increase digital functionality may increase cost Practical—yes Acceptable—yes, however, further piloting of online activities would be necessary to confirm
Online-based self-completed session (Module Session 1); • Pedometer for tracking daily footsteps on personal page of online-based programme • Nurses requested to keep a record of unhealthy snacking/physical inactivity on personal page of online-based programme	Digital—individual	Affordable—yes, however increase digital functionality may increase cost Practical—yes Acceptable—yes, however, further piloting of online activities would be necessary to confirm
Online-based self-completed session (Module Session 2); • Invite nurses to undertake a fitness and strength test	Digital—individual	Affordable—yes, however increase digital functionality may increase cost Practical—yes Acceptable—yes, however, further piloting of online activities would be necessary to confirm
A brief message about the increased risk of chronic disease following unhealthy dietary/physical activity patterns placed on pedometer in conjunction with advice on how to mitigate this risk	Printed material	Affordable—yes Practical—yes Acceptable —yes
Online-based self-completed session (Module Session 2); • Nurses requested to keep a record of unhealthy snacking/physical inactivity on personal page of online-based programme. This is to identify situations or events occurring prior to unhealthy snacking/physical inactivity.	Digital—individual	Affordable—yes, however increase digital functionality may increase cost Practical—yes Acceptable—yes, however, further piloting of online activities would be necessary to confirm
Online group-based motivational interviewing session (Module Session 3); • A registered dietitian/nutritionist/physiotherapist provides feedback within motivational interviewing session on healthy eating/physical activity progress.	Digital—grouped	Affordable—yes, however increase digital functionality and ongoing employment of healthcare professionals may increase cost Practical—yes Acceptable—yes, however, further piloting of online activities would be necessary to confirm
Online group-based motivational interviewing session (Module Session 3); • Nurses verbally persuaded to enhance self-efficacy as part of online group-based motivational interviewing session	Digital—grouped	Affordable—yes, however increase digital functionality may increase cost Practical—yes Acceptable—yes, however, further piloting of online activities would be necessary to confirm acceptability
Online-based self-completed session (Module Session 2) Nurses advised to track their mood alongside their eating and physical activity behaviours on personal page of online-based programme. This is to support nurses identify the emotional consequences of healthy and unhealthy behaviours for themselves.	Digital—individual	Affordable—yes, however increase digital functionality may increase cost Practical—yes Acceptable—yes
Online-based self-completed session (Module Session 2) Nurses requested to keep a record of the dangers of eating unhealthy/being sedentary on personal page of online-based programme. Concrete information on what will happen if a nurse does this every day (e.g. if you eat like this every day for the next 4 weeks you will lose/gain X kg) provided to increase the personal salience of the consequences of unhealthy behaviours.	Digital—individual	Affordable—yes, however increase digital functionality may increase cost Practical—yes Acceptable—yes
Online group-based motivational interviewing session (Module Session 3) Nurses requested to answer the following two questions (“If I did not eat healthily and participate in physical activity I would later feel regret.”) and (“If I did not eat healthily and participate in physical activity, I would later wish I had.”)	Digital—grouped	Affordable—yes, however increase digital functionality may increase cost Practical—yes Acceptable—yes
A web-based staff newsletter and social media used for communicating information about social and environmental consequences of healthy eating and physical activity	Digital—individual	Affordable—yes, however increase digital functionality may increase cost Practical—yes Acceptable—yes
Online-based self-completed session (Module Session 2) Prompt nurses to imagine and compare likely or possible outcomes following eating healthily/participating in physical activity versus not performing these behaviours.	Digital—individual	Affordable—yes, however increase digital functionality may increase cost Practical—yes Acceptable—yes
Online group-based motivational interviewing session (Module Session 3) A registered dietitian/nutritionist/physiotherapist would provide feedback on healthy eating/physical activity progress.	Digital—grouped	Affordable—yes, however increase digital functionality and ongoing employment of healthcare professionals may increase cost Practical—yes Acceptable—yes
A fear appeal message in combination with a self-affirmation message incorporated into social marketing campaign	Digital—grouped	Affordable—yes Practical—yes Acceptable—no, however confirmation of fear appeals acceptability would need to be tested
Online-based self-completed session (Module Session 4) Nurses requested to list and compare the advantages and disadvantages of eating healthy/physical activity participation	Digital—individual	Affordable—yes, however increase digital functionality may increase cost Practical—yes Acceptable—yes
Online-based self-completed session (Module Session 4) Expert video discussing the use of stress management techniques such as progressive muscular relaxation and diaphragmatic breathing to help nurses manage their stress	Digital—individual	Affordable—yes, however increase digital functionality may increase cost Practical—yes Acceptable—yes
Online-based self-completed session (Module Session 4) Advises nurses to record how they feel after eating healthily/participating in physical activity on personal page of online-based programme	Digital—individual	Affordable—yes, however increase digital functionality may increase cost Practical—yes Acceptable—yes
Online-based group-based forum and discussion (Module Session 5) Participants could share experiences and give each other tips or search for training partners.	Digital—grouped	Affordable—yes, however increase digital functionality may increase cost Practical—yes Acceptable—yes, however, options for anonymously sharing experiences would need to be considered

## **Discussion**

The current study describes the systematic, evidence-based and theory-informed approach to developing a tailored workplace intervention that aims to change nurses’ eating and physical activity behaviours. The intervention has been rigorously developed to reflect current best practice in intervention development [20, 23, 27]. It is anticipated that by adopting a systematic, evidence-based and theory-informed approach for intervention development, the capacity to effectively change nurses’ eating and physical activity behaviours will be improved. To change nurses’ eating and physical activity outcomes, the intervention is specified by nine intervention functions and seven policy categories and consists of 22 evidence-based and theoretically underpinned BCTs.

It is unclear at this stage whether the depicted scenario is superior to other potential options. A digital mode of delivery of BCTs targeting individual level determinants of eating and physical activity behaviours is expected to enhance the potential for equitable intervention delivery. Nurses’ values and preferences should also be used to guide decisions around what workplace intervention mode of delivery to take forward. Adopting a person-centred approach and basing the intervention development on nurses’ views is anticipated to enhance the likelihood that the intervention will be accepted and ultimately effective [33]. Evidence generated from our formative work indicates that approximately three in every four nurses would be willing in principle to participate in a randomised controlled trial of a workplace eating and physical activity intervention. Improving participation among nurses is particularly important given reports that of all healthcare professional groups working in hospitals, nurses have the lowest participation in workplace health promotion activities, despite displaying the highest rates of obesity and overweight [3].

Several aspects of the developed intervention are innovative. For example, few studies have proposed to change nurses’ beliefs about consequences. The intervention will enable nurses to adopt comparative imagining of future outcomes (in the form of online group-based motivational interviewing sessions), an effective technique for changing eating and physical activity behaviours [34, 35]. Additionally, the intervention will use social support to change emotional responses. It is widely recognised that for initiating and sustaining health behaviour change, positive types of social support from friends, family and colleagues is particularly important [36]. Online (in the form of an online support community tailored to the preferences and characteristics of nurses specifically) and face-to-face sources of social support both appear to help people maintain long-term health behaviour changes [37].

To ensure implementation success, consideration should also be given to the affordability, practicability, effectiveness/cost-effectiveness, acceptability, side-effects/safety and equality (APEASE) of the intervention. By using this APEASE criteria (Table 5) [23], additional insights can be made to inform a tailored intervention package and implementation strategy. For instance, implementing many of the environmental changes will require large numbers of stakeholders at multiple levels of influence to also change their behaviour. Reluctance or inability of stakeholders to introduce environmental changes may limit the feasibility, acceptability and practicality of implementing environmental changes. Assessing the extent to which stakeholders such as hospital chief executives and department managers would be supportive of potential changes to the hospital environment is an important step in the development of an effective intervention implementation strategy [12]. As they are likely to be the agents who will decide on implementation options, an assessment of their views may provide an avenue for enhancing their ‘buy-in’ and advocacy.

## Strengths and limitations

The present study applied a theoretical and evidence-based approach to behaviour change. Determinants of two target behaviours (i.e. eating and physical activity) were identified and mapped to appropriate intervention functions, policy categories and BCTs to develop a specified workplace behaviour change intervention tailored for nurses. In line with dual-process or dual-systems theories of behaviour [38], it is important to highlight that the determinants identified within this study represent the perceptions of nurses and as such reflect only those that operate with some degree of conscious awareness. There may be additional determinants shaping nurses’ eating and physical activity behaviour, beyond their awareness that have not been captured (e.g. unconscious biases towards eating behaviours or activities). The selection of intervention functions and policy categories was undertaken in the intervention development process to facilitate a precisely specified and parsimonious intervention. In retrospect, this step in the development process was less directive than expected. It was found that any proposed intervention might incorporate all the available intervention functions and policy categories. This was the first application of the BCW and TDF together to the development of a workplace eating and physical activity behaviour change intervention. Hence, deciding on the most appropriate intervention functions and policy categories in the context of limited prior guidance available may be one reason for the lack of brevity.

An alternative and perhaps more useful approach would have been to proceed directly from the behavioural diagnosis using the TDF (steps 1 and 2) to selecting BCTs for the intervention. Notwithstanding this, by reporting the intervention development steps completely and transparently, hypothesised mechanisms of behaviour change in any resulting intervention could be tested in a definitive clinical trial [39]. Such sharing of best practice in intervention development can contribute to a cumulative understanding of eating and physical activity behaviour change in nurses, thereby increasing the value of this research and the proposed interventions replication potential [27]. The methods used for operationalising BCTs were subjective. It is therefore possible that another research team would yield different ideas for how best to operationalise the BCTs. Standardised and transparent methods to report BCT operationalisation will need to be established to ensure this phase of the intervention development process can be replicated.

### Implications for research

Given the increased scrutiny being placed on the ease with which published results can be reproduced or replicated [40], the intervention development methods presented in this study represent an important contribution to knowledge. They have the potential to avoid research redundancy. An exploration of nurses’ preferences for the best modes of delivery for these BCTs (e.g. face-to-face such as one-on-one or group, print or web) and intervention intensity (e.g. contact frequency, number of contacts, contact time) is warranted to further optimise the intervention development process.

### Implications for practice

The workplace intervention developed in this study is now ready for formal evaluation in a trial and might have the potential in future to markedly impact upon employee absenteeism, presenteeism, productivity and retention outcomes [41, 42] and overall quality of care [14]. Highlighting the possible financial returns for employers on investment in health interventions may serve to increase uptake of the developed workplace intervention among healthcare organisations. The intervention meets expert recommendations on promoting employee health in the UK National Health Service system [36]. It therefore represents a practical application through which healthcare organisations can improve nurses’ eating and physical activity practices.

## Conclusions

The current study presents a detailed example of how a workplace behaviour change intervention can be developed by integrating empirical evidence, with the TDF and BCTs. In this sense, it will enable research evidence to accumulate on the content used, which should inform any potential replication efforts in the future. The cohesive workplace intervention developed provides a prototype which could be taken forward for full feasibility testing and formal evaluation.

## Supplementary Information

**Additional file 1.** Template for intervention description and replication (TIDieR) checklist.

**Additional file 2.** Evidence of BCT effectiveness and parameters for BCT effectiveness.

## Data Availability

The datasets collected and/or analysed during the current study are available from the corresponding author on request.

## Abbreviations

UK: United Kingdom
MRC: Medical Research Council
TDF: Theoretical Domains Framework
BCW: Behaviour Change Wheel
BCTTv1: Behaviour change techniques taxonomy version
BCT: Behaviour change technique
TIDieR: Template for Intervention Description and Replication
APEASE: Affordability, practicability, effectiveness/cost-effectiveness, acceptability, side-effects/safety and equality
