# Supplementary material for: Development of a behaviour change workplace-based intervention to improve nurses’ eating and physical activity
Source: Pilot Feasibility Stud. 2021 Feb 18;7:53. doi: 10.1186/s40814-021-00789-0 (PMC7891147; doi:10.1186/s40814-021-00789-0)
Supplement: Supplementary file 1 — Additional file 1. Template for intervention description and replication (TIDieR) checklist. [file 40814_2021_789_MOESM1_ESM.zip › Additional File 1 TIDIER Reporting Checklist B POWER V2R1.docx]

**Additional file 1**

**Completed TIDieR checklist**


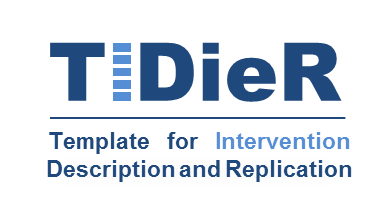


**The TIDieR (Template for Intervention Description and Replication) Checklist**

Information to include when describing an intervention and the location of the information

| **Item number** | **Item** | **Details**  **Primary paper (page or appendix number)** |
| --- | --- | --- |
|  |  |  |
|  | **BRIEF NAME** |  |
| **1.** | Provide the name or a phrase that describes the intervention. | Workplace-based eating and physical activity intervention |
|  | **WHY** |  |
| **2.** | Describe any rationale, theory, or goal of the elements essential to the intervention. | The workplace intervention was developed using the using a Theoretical Domains Framework, Behaviour Change Wheel and Behaviour Change Technique Taxonomy and aims to improve nurses’ eating and physical activity behaviours |
| **Item number** | **Item** | **Details**  **Primary paper (page or appendix number)** |
|  | **WHAT** |  |
| **3.** | Materials: Describe any physical or informational materials used in the intervention, including those provided to participants or used in intervention delivery or in training of intervention providers. Provide information on where the materials can be accessed (e.g. online appendix, URL). | The workplace intervention includes:  (1) Online programme delivery of behaviour change techniques implemented across five modules. Two online modules; module sessions three and five will be group-based and will be delivered by dietetic/ registered nutritionist and physiotherapy staff online. Three further online modules will be individualised and self-completed by nurses.  (2) Food Labelling and Vinyl Footsteps placed at hospital sites  (4) Pedometers  (5) Diary for self-monitoring within personal page of online programme  (6) Online newsletter and social media (twitter) for message dissemination |
| **4.** | Procedures: Describe each of the procedures, activities, and/or processes used in the intervention, including any enabling or support activities. | The intervention delivers 22 BCTs to nurses by a combination of digital and printed materials. Dietetics/ registered nutritionist and physiotherapy staff will deliver group-based online motivational interviewing and provide feedback and vicarious reinforcement to nurses during module sessions three and four of the online programme.  A pedometer and online diary will be provided to nurses with information on tracked behaviour changes throughout the intervention.  Vinyl footsteps and food labelling will be displayed in hospitals to cue nurses to choose physical activity and healthy eating options. |
| **Item number** | **Item** | **Details**  **Primary paper (page or appendix number)** |
|  | **WHO PROVIDED** |  |
| **5.** | For each category of intervention provider (e.g. psychologist, nursing assistant), describe their expertise, background and any specific training given. | Eligibility criteria for staff delivering module sessions within the online programme:   1. Be state registered as a dietitian with the national regulatory body 2. Be registered as a registered nutritionist with a national association 3. Be state registered as a physiotherapist with the national regulatory body 4. Be experienced (2 years’ experience) in motivational interviewing 5. Be able to advise on acceptability or feasibility issues with the intervention and study protocol.   The online programme deliverers above will be briefed by the research team and provided with the online session materials for presentation to nurses.  A gate keeper will be identified at the participating hospitals and invited to identify appropriate staff members to deliver the online module sessions. |
| **Item number** | **Item** | **Details**  **Primary paper (page or appendix number)** |
|  | **HOW** |  |
| **6.** | Describe the modes of delivery (e.g. face-to-face or by some other mechanism, such as internet or telephone) of the intervention and whether it was provided individually or in a group. | The intervention will be delivered via an online platform using five pre-developed modules. Two module sessions (module sessions three and five) will be group-based and delivered by dietetic/ registered nutritionist and physiotherapy staff. The other three modules will be self-completed individually by nurse participants. The printed materials of the intervention will be implemented in the hospital canteen and at stairways. |
|  | **WHERE** |  |
| **7.** | Describe the type(s) of location(s) where the intervention occurred, including any necessary infrastructure or relevant features. | The intervention will be delivered at hospital sites, with online access also available outside of nurse participants’ place of work. |
|  | **WHEN and HOW MUCH** |  |
| **8.** | Describe the number of times the intervention was delivered and over what period of time including the number of sessions, their schedule, and their duration, intensity or dose. | The workplace intervention will be delivered in sequence over 12 months. Three individually self-completed online sessions will last approximately 30 minutes each at first sitting. Nurses will then have access to these three self-completed module sessions over a 12 month period. The two group-based online module sessions will be accessible every three months per group over a period of 12 months (4 sessions in total for each group). The printed materials of the workplace intervention would be in place for the entire duration of the intervention (12 months). |
| **Item number** | **Item** | **Details**  **Primary paper (page or appendix number)** |
|  | **TAILORING** |  |
| **9.** | If the intervention was planned to be personalised, titrated or adapted, then describe what, why, when, and how. | The intervention is tailored to nurses’ demographic and behavioural characteristics to increase the intervention contents  personal relevance and hence engagement among nurses. The intervention may be adapted further prior to implementation. |
|  | **MODIFICATIONS** |  |
| **10.^ǂ^** | If the intervention was modified during the course of the study, describe the changes (what, why, when, and how). | N/A (intervention not yet delivered). |
|  | **HOW WELL** |  |
| **11.** | Planned: If intervention adherence or fidelity was assessed, describe how and by whom, and if any strategies were used to maintain or improve fidelity, describe them. | Fidelity of intervention delivery will be assessed using log-ins for online module sessions, module session deliverers’ reports and intervention exit qualitative interviews. |
| **12.^ǂ^** | Actual: If intervention adherence or fidelity was assessed, describe the extent to which the intervention was delivered as planned. | N/A (intervention not yet delivered). |

** **Authors** - use N/A if an item is not applicable for the intervention being described. **Reviewers** – use ‘?’ if information about the element is not reported/not sufficiently reported.

ǂ If completing the TIDieR checklist for a protocol, these items are not relevant to the protocol and cannot be described until the study is complete.
* We strongly recommend using this checklist in conjunction with the TIDieR guide (see *BMJ* 2014;348:g1687) which contains an explanation and elaboration for each item.

* The focus of TIDieR is on reporting details of the intervention elements (and where relevant, comparison elements) of a study. Other elements and methodological features of studies are covered by other reporting statements and checklists and have not been duplicated as part of the TIDieR checklist. When a **randomised trial** is being reported, the TIDieR checklist should be used in conjunction with the CONSORT statement (see [www.consort-statement.org](http://www.consort-statement.org)) as an extension of **Item 5 of the CONSORT 2010 Statement.** When a **clinical trial** **protocol** is being reported, the TIDieR checklist should be used in conjunction with the SPIRIT statement as an extension of **Item 11 of the SPIRIT 2013 Statement** (see [www.spirit-statement.org](http://www.spirit-statement.org)). For alternate study designs, TIDieR can be used in conjunction with the appropriate checklist for that study design (see [www.equator-network.org](http://www.equator-network.org)).
