# Supplementary material for: Development of a behaviour change workplace-based intervention to improve nurses’ eating and physical activity
Source: Pilot Feasibility Stud. 2021 Feb 18;7:53. doi: 10.1186/s40814-021-00789-0 (PMC7891147; doi:10.1186/s40814-021-00789-0)
Supplement: Supplementary file 2 — Additional file 2. Evidence of BCT effectiveness and parameters for BCT effectiveness. [file 40814_2021_789_MOESM2_ESM.docx]

Additional file 2

Evidence of BCT effectiveness and parameters for BCT effectiveness

Scoping Review Search Strategy

Medline database was searched for the scoping review. The search terms used included: ‘intervention?.tw.’, ‘BCT*’, ‘behavio* change strateg*’, ‘behavio* change technique*’, ‘((increas* or improv*) adj1 (fitness or physical activity)).tw.’, ‘(dietary adj1 (intake or behavio*r*)).tw.’, ‘Obesity/’, ‘Overweight/’, ‘exp body weight changes/’, ‘(weight adj1 (loss or lose or losing or lost or maintain* or reduc* or decreas* or chang*)).tw.’, ‘(systematic adj review).tw’, ‘review.tw, ‘(meta adj analysis).tw’ meta-analysis.tw’. Reference lists of retrieved systematic reviews were also assessed for additional literature. The Medline database search was limited to adults and English language articles.

The selected BCTs, how they may possibly be operationalised, evidence for their effectiveness and their parameters for effectiveness are detailed below.

***Environmental context and resources – TDF domain identified as important barrier***

*BCT to change this domain: 1 Restructuring the physical environment*

To change nurses’ eating behaviour, restructuring the physical environment might be operationalised by providing food storage for unhealthy snacks that is located somewhere less accessible and more inconvenient to get to. Modification of the nutritional profile of food and beverages sold from vending machines in workplaces might also be undertaken. As an example, increase the ratio of healthy foods and beverages to unhealthy foods and beverages with low nutritional value stocked in vending machines. Further ways to operationalise this BCT include; nutrition labelling, changing canteen food supply/availability, menu reformulation and providing information and reducing the availability of unhealthy foods in the environment. For changing nurses’ physical activity behaviour, operationalisation might involve planning local facilities and services to contain clear and legible foot paths and cycle paths to make active travel to and from work easier. Another approach to consider would be to increase the availability and accessibility of workplace quality leisure facilities (e.g. gyms, pools and showering facilities) and water coolers. Given that lack of access to portable drinking water was regarded by nurses as an important determinant of their eating and physical activity behaviours, using water coolers is a particularly crucial operationalisation. Sit-stand workstations (i.e. height-adjustable desk equipment e.g. [www.ergotron.com](http://www.ergotron.com)) might also be implemented at nurses’ stations. A number of systematic reviews have demonstrated that restructuring the physical environment is effective in changing eating and physical activity behaviours (Skov *et al*., 2013; Arnott *et al*., 2014; Grech and Allman-Farinelli, 2015). Contrastingly, there is a lack of evidence that this BCT is ineffective or worsens eating and physical activity behaviours. In relation to parameters for BCT effectiveness, it should be noted that nurses with high self- efficacy for physical activity or who perceive strong social support or social pressure to be physical active, might be less influenced by restructuring the physical environment than those with lower levels of such factors (Mackenbach *et al*., 2014).

*BCT to change this domain: 2. Discriminative (learned) cue (cue signalling reward)*

Discriminative (learned) cue (cue signalling reward) could be operationalised through advising nurses that a non-monetary incentive would be provided monthly for taking the stairs during the working day but not the lift to encourage taking the stairs (the stairs is the environmental stimulus). According to Deckersbach *et al*. (2014) and Rimm *et al*. (2015) discriminative (learned) cue (cue signalling reward) is effective in changing eating and physical activity behaviours respectively. On the other hand, there is a lack of evidence that discriminative (learned) cue (cue signalling reward) is ineffective or worsens eating and physical activity behaviours.

*BCT to change this domain: 3. Prompts/cues*

Prompts/cues might be operationalised with point-of-choice food labelling (e.g. reference portion size labelling), motivational prompts (signs, posters, footprint symbols) for stair versus lift or escalator use and promotional signage and materials including posters, screens, audio, public announcements. Further to this, placing vinyl footsteps on floors indicating the path to the stairs might also be used as prompts to promote stair use in the workplace. Operationalising prompts/cues through smartphone applications such as RunKeeper, Weight Watchers Mobile or Couch-to-5K are additional approaches that should be considered. It is worth highlighting that much evidence to support the role of prompts/cues in changing eating and physical activity behaviours exists (Soler *et al*., 2010; Kremers, Eves and Andersen, 2012; Olander *et al*., 2013; Lara *et al*., 2014; National Institute for Health and Clinical Excellence, 2014; Schüz, Bower and Ferguson, 2015). However, it is notable that Hankonen *et al.* (2014) reported that prompts/cues in the form of placing action plans on a fridge door were ineffective in changing eating and physical activity behaviours. The following parameters for prompts/cues effectiveness should be considered; (i) the prompt or cue should normally occur at the time or place of performance of the target behaviour (ii) interactions between prompts/cues and participant characteristics and between prompts/cues and intervention context (e.g. stairs versus escalators, or stairs versus lifts) (Lewis and Eves, 2011; Lewis and Eves, 2012). That is, when using prompts/cues to change eating and physical activity behaviours, attention should be paid to nurses’ characteristics (e.g. weight status) and the context (e.g. workplace building layout) to improve prompt/cue effectiveness, (iii) frequency and tailoring (Fry and Neff, 2009) and (iv) point of choice food labelling more effective when using nutrition information that is liked by target population (Hoefkens *et al*., 2011).

*BCT to change this domain: 4. Restructuring the social environment*

The organisation of workplace activity groups (Hanson and Jones, 2015) could be an approach taken to operationalise restructuring the social environment. Another way to operationalise restructuring the social environment would be to develop social norm messages via a social marketing campaign (Robinson *et al*., 2014). With regard to evidence of effectiveness, Fortier *et al*. (2012) and Hollands *et al*. (2013) report that restructuring the social environment can be effective in changing eating and physical activity behaviours. By contrast, there is a lack of evidence that restructuring the social environment is ineffective or worsens eating and physical activity.. Similarly, there is a lack of evidence on parameters for BCT effectiveness.

*BCT to change this domain: 5. Avoidance/changing exposure to cues for the behaviour*

In the case of avoidance/changing exposure to cues for the behaviour, advising nurses to pack healthy options daily such as fresh fruit instead of chocolate or crisps for work is an approach that could be used. Other potential approaches to operationalise this BCT include advising nurses to move a tin left by patients’ relatives that contains chocolates and sweets from the nursing desk on the ward to a cupboard in the kitchen, if they usually eat these chocolates and sweets. Further ways in which this BCT could be operationalised include recommending nurses to do the following (i) when at a social occasion to put themselves out of reach of tempting foods (Poelman *et al*., 2014); (ii) when eating foods (either in workplace/out of workplace), determine normal serving in advance, and store the rest out of sight and reach (Poelman *et al*., 2014) and (iii) advise nurses to focus on activities other than watching television and going to pubs and bars (whichever applicable) that have been associated with previous sedentary behaviour. Hollands *et al*. (2013) report that this BCT is effective in changing eating and physical activity behaviours. There is a lack of evidence that BCT ineffective or worsens eating and physical activity behaviours.. Further to this, there is a lack of evidence on parameters for effectiveness for this BCT..

***Knowledge – TDF domain identified as important enabler***

*BCT to change this domain: 1. Health consequences*

Explaining to nurses the increased risk of cardiovascular disease/cancer following unhealthy dietary/physical activity patterns via automated mobile phone text-messages (Hutchesson *et al*., 2015) in conjunction with advice on how to mitigate this risk is one way to operationalise the BCT, information on health consequences. It has been demonstrated that providing information on health consequences can be effective in changing eating and physical activity (Bird *et al*., 2013; Olander *et al*., 2013; van Vugt *et al*., 2013; Arnott *et al*., 2014; Lara *et al*., 2014; Lyons *et al*., 2014). No evidence demonstrating that this BCT is ineffective in changing eating and physical activity was retrieved. However, French *et al*. (2014) reported evidence that the provision of information on health consequences may actually worsen eating and physical activity behaviours. Moreover, parameters for effectiveness to remain cognisant of include; (i) consequences can be for any target, not just the recipient (s) of the intervention and (ii) providing information on health consequences only motivates behaviour change when accompanied with active, BCTs that outline how to mitigate the identified risk (Albarracin *et al*., 2005).

*BCT to change this domain: 2. Biofeedback*

Concerning the operationalisation of biofeedback, nurses could be requested to monitor their blood glucose on a portable blood glucose self-monitoring device (Campfield and Smith, 2003; Ciampolini and Bianchi, 2006; Jospe *et al*., 2015) and instructed to eat only when physical hunger confirmed (blood glucose in specific target range) to improve healthy eating behaviours. Using a portable pedal machine biofeedback display on blood pressure to encourage nurses to adopt light-intensity physical activity (i.e. active sitting) throughout the day and undertaking a fitness and strength test via website are other potential operationalisation approaches for biofeedback. Active sitting is defined as increased trunk movement to maintain an upright sitting posture by an individual through the use of an unstable seating surface. In addition, exercise heart rate monitors could be used by nurses to provide feedback on physical activity intensity as reflected by heart rate. Hansen *et al*. (2012); Carr *et al*. (2013) and Teufel *et al*. (2013) all found biofeedback as an effective BCT for changing eating and physical activity behaviour. In addition to this, there is a lack of evidence that BCT is ineffective in changing eating and physical activity or worsens eating and physical activity behaviours .

*BCT to change this domain: 3. Antecedents*

Advising nurses to keep a record of unhealthy snacking/physical inactivity and the immediately preceding events would allow them to identify likely antecedents to unhealthy snacking/physical inactivity. The effectiveness of antecedents in changing eating and physical activity is inconsistent. For example, Hunt *et al*. (2014) found antecedents (Identifying and drawing awareness to them, and identifying and advising to change them) effective in changing eating and physical activity, while in a separate systematic review Hartmann-Boyce *et al*. (2014) reported antecedents as ineffective in changing eating and physical activity behaviour. There is a lack of evidence that this BCT worsens eating and physical activity or on parameters for antecedents’ effectiveness.

*BCT to change this domain: 4. Feedback on behaviour*

Operationalising the BCT feedback on behaviour might involve informing a nurse of how many steps they walked each day (as recorded on a pedometer/ accelerometer) or how many calories they ate each day (based on a food record). Other recommended approaches to operationalise feedback on behaviour include using a self-administered, web-based programme or smartphone application to provide feedback on eating and physical activity Analysis of the evidence base reveals a mixed picture for the effectiveness of providing feedback on behaviour in changing eating and physical activity. For instance, reviews carried out by Bird *et al*. (2013); Lyons *et al*. (2014); Olander *et al*. (2013) and Tang *et al*. (2014) found evidence that providing feedback on behaviour is effective in changing eating and physical activity behaviours. However, Hartmann-Boyce *et al*. (2014) reported this BCT is ineffective in changing eating and physical activity behaviours, whilst French *et al*. (2014) reported this BCT even worsens eating and physical activity behaviours. With regard to parameters for feedback on behaviour effectiveness, feedback as a BCT is more likely to be effective if the feedback is personalised, follows the target behaviour closely in time, and is specific (Kazdin *et al*., 2008). The use of self-monitoring of behaviour and subsequent feedback is also a typically effective combination (Abraham and Michie, 2008; Greaves *et al*., 2011).

***Optimism – TDF domain identified as important enabler***

*BCT to change this domain: 1. Verbal persuasion to boost self-efficacy*

To operationalise the BCT verbal persuasion to boost self-efficacy, motivational interviewing which aims to evoke self-efficacy to change could be considered. Searching the evidence base, Hollis *et al.* (2012) and French *et al*. (2014) found that motivational interviewing was not effective in changing eating and physical activity behaviours. Contrastingly, a recent UK weight loss maintenance feasibility trial found motivational interviewing potentially effective (Simpson *et al*., 2015). However, the limited evidence base on the ‘active ingredients’ of motivational interviewing makes it difficult to draw conclusions about the specific BCTs by which motivational interviewing changes eating and physical activity behaviours (Dobber *et al*., 2015).

***Beliefs about consequences – TDF domain identified as important enabler***

*BCT to change this domain: 1. Emotional consequences*

Operationalising emotional consequences might include advising nurses to track their mood alongside their eating and physical activity behaviours so they can see the emotional consequences of healthy and unhealthy behaviours for themselves. With respect to evidence of effectiveness, a systematic review by Lyons *et al*. (2014) found that changing emotional consequences was effective in improving both eating and physical activity. When considering BCT ineffectiveness and evidence of this BCT worsening eating and physical activity there is a lack of evidence. Additionally, there is a lack of evidence on parameters for BCT effectiveness.

*BCT to change this domain: 2. Salience of consequences*

For salience of consequences, producing material to clearly illustrate health consequences e.g. the risk of becoming overweight, highlighting the dangers of continuing to eat unhealthily/be sedentary via written material or smartphone application are options to consider. Further, concrete information on what will happen if a nurse does this every day (e.g. if you eat like this every day for the next 4 weeks you will lose/gain X Kgs) could be provided to increase the personal salience of the consequences of unhealthy behaviours. The National Institute for Health and Clinical Excellence (2014) and Allan *et al*. (2015) suggest salience of consequences is effective in improving eating and physical activity. There is a lack of evidence to indicate that salience of consequences is ineffective or worsens eating and physical activity behaviours’. Additionally, there is a lack of evidence on parameters for BCT effectiveness.

*BCT to change this domain: 3. Covert sensitisation (Imaginary punishment)*

Studies on covert sensitisation are limited to the 1970’ and 1980’s. Approaches used to operationalise this BCT in the literature include advising individuals to imagine overeating or being physical inactive and then becoming ill. Janda and Rimm (1972) found covert sensitisation to be effective in changing eating and physical activity. Evidence that this BCT is ineffective in changing eating and physical activity was reported by Little (1978). By contrast, there was a lack of evidence retrieved suggesting covert sensitisation worsens eating and physical activity behaviours. There is also a lack of evidence on parameters for BCT effectiveness.

*BCT to change this domain: 4. Anticipated regret*

In operationalising anticipated regret, asking nurses to assess the degree of regret they will feel if they do not eat healthily or engage in physical activity could be considered. There is evidence supporting the role of anticipated in predicting eating behaviour change (Steptoe *et al*., 2004), strengthening intentions to eat healthily (Sandberg and Conner, 2008; Weijzen, de Graaf and Dijksterhuis, 2009; Godin *et al*., 2010) and increasing physical activity intentions and behaviour (Abraham and Sheeran, 2004; Sandberg and Conner, 2011). On the other hand, Hartmann-Boyce *et al*. (2014) reported anticipated regret as ineffective in changing eating and physical activity. There is a lack of evidence that this BCT worsens eating and physical activity behaviours or parameters for BCT effectiveness.

*BCT to change this domain: 5. Social and environmental consequences*

For this BCT, operationalisation might include the use of a smartphone application such as jawbone. Jawbone is an electronic activity monitor that provides interactive behaviour change tools via a mobile device or personal computer. An investigation of the behavioural change literature identified a systematic review by Lyons *et al*. (2014) which supported the use of social and environmental consequences to change eating and physical activity. There is a lack of evidence that this BCT is ineffective or worsens eating and physical activity. Further to this, there is a lack of evidence on parameters for BCT effectiveness.

*BCT to change this domain: 6. Comparative imagining of future outcomes*

Prompting a nurse to imagine and compare likely or possible outcomes following eating healthily/participating in physical activity versus not performing these behaviours via motivational interviewing is one potential approach to operationalise this BCT. Armstrong *et al*. (2011); Morton *et al*. (2014) and O’Halloran *et al*. (2014) all provide evidence that comparative imagining of future outcomes (in the form of motivational interviewing) is effective in changing eating and physical activity. Contrastingly, there is a lack of evidence that this BCT is ineffective or makes eating and physical activity behaviours worse. Similarly, in relation to parameters of effectiveness there is a lack of evidence.

*BCT to change this domain: 7. Vicarious reinforcement*

Operationalising vicarious reinforcement might involve drawing attention to the positive comments other staff get when they eat healthily/participate in physical activity regularly. Based on best available evidence (Valente *et al*., 2007; Fox and Bailenson, 2009), vicarious reinforcement appears to be effective in changing eating and physical activity. There is a lack of evidence to suggest that vicarious reinforcement is ineffective or worsens eating and physical activity. To date, there is a lack of evidence on parameters of effectiveness for vicarious reinforcement.

*BCT to change this domain: 8. Threat (future punishment) may include fear arousal*

Operationalising this BCT might include informing a nurse that continuing to eat unhealthily and remaining physically inactive is likely to result in the risk of a heart attack or premature mortality. Findings that threat is effective in changing eating and physical activity behaviour have been reported by Ritland and Rodriguez (2014). However, in a systematic review Hartmann-Boyce *et al*. (2014) threat was found to be ineffective in changing eating and physical activity. Taking parameters for effectiveness into account, it is important to note that the nurse should perceive the threat as severe and to be at risk in order for the threat to produce behaviour change. A nurse also needs to believe that the recommended behaviour is effective to avert the threat and possess high self-efficacy to perform the target behaviour. A further parameter for effectiveness to consider is that self-affirmation may be used to reduce defensive message processing and promote open-minded appraisal of health risk information (Epton and Harris, 2008; Epton *et al*., 2015). Self-affirmation inductions such as writing exercises that remind participants of their important personal values is one technique that can be used to self-affirm individuals. For example, Epton and Harris (2008) asked participants to recall and give examples of past acts of kindness with questions such as, “Have you ever forgiven another person when they have hurt you? yes-no,” and “Have you ever been considerate of another person's feelings? yes-no.” to self-affirm participants. When the reply to these questions was “yes,” participants were then required to give examples of their behaviour. Recent evidence also reports fear message with non-indulgent food offerings interact to determine an exercise goal (Krishen and Bui, 2015). The importance of these parameters for BCT effectiveness is highlighted by Witte *et al*. (2000) who found that dispensing threats including fear arousal may make health behaviours such as eating and physical activity worse if parameters for effectiveness are not included (Witte and Allen*,*  2000). Peters, Ruiter and Kok (2013) also report that under conditions of low efficacy, fear appeals can lead individuals to engage in unhealthy behaviours.

*BCT to change this domain: 9. Pros and cons*

Operationalisation of this BCT might involve advising the nurse to list and compare the advantages and disadvantages of eating healthily/physical activity participation via motivational interviewing. Evidence that the BCT is effective in changing eating and physical activity has been reported by Hardcastle *et al*. (2008) and Arnott *et al*. (2014). On the other hand, there is a lack of evidence that this BCT is ineffective or worsens eating and physical activity. Likewise, when considering parameters for BCT effectiveness there is a lack of evidence.

*BCT to change this domain: 10. Covert conditioning (imaginary reward)*

The evidence base on covert conditioning primarily focusses on pain, phobias, asthma, depression and drug addiction. From this evidence base, appropriate operationalisation might include requesting the nurse to imagine performing eating healthily/participating in physical activity and follow it with a pleasant image. Nurses might also actually perform these health behaviours followed by an imagined reward or imagine performing these health behaviours followed by receipt of an actual reinforcer. There is a lack of evidence that this BCT is effective in changing eating and physical activity behaviour, or makes eating and physical activity behaviours worse. There is also a lack ofevidence on parameters for BCT effectiveness.

***Emotion – TDF identified as important barrier***

*BCT to change this domain: 1. Reduce negative emotions (includes stress management)*

For this BCT, operationalisation might include advising nurses on the use of stress management techniques such as progressive muscular relaxation and diaphragmatic breathing in small group meetings or on individual basis or using cognitive behavioural therapy. Evidence that reducing negative emotions is effective in changing eating and physical activity behaviours has been reported in several studies (Manzoni *et al*., 2009; Webb *et al*., 2010; Christaki *et al*., 2013). By contrast, Katzer and Bradshaw (2008) found reducing negative emotions is ineffective in changing eating and physical activity. There is a lack of evidence that this BCT worsens eating and physical activity or parameters for BCT effectiveness.

*BCT to change this domain: 2. Emotional consequences*

As already discussed, operationalising emotional consequences might include advising nurses to track their mood alongside their eating and physical activity behaviours so they can see the emotional consequences of healthy and unhealthy behaviours for themselves. With respect to evidence of effectiveness, a systematic review by Lyons *et al*. (2014) found that changing emotional consequences was effective in improving both eating and physical activity. When considering BCT ineffectiveness and evidence of BCT worsening eating and physical activity there is a lack of evidence. Additionally, there is a lack of evidence on parameters for BCT effectiveness.

*BCT to change this domain: 3. Self-assessment of affective consequences*

Operationalising self-assessment of affective consequences might incorporate the setting of an agreement with nurses that they record how they feel after eating healthy/taking their daily walk combined with feedback and monitoring. Notably, Pfattheicher and Sassenrath (2014) found self-assessment of affective consequences to be effective in changing eating behaviour. A search for evidence that self-assessment of affective consequences is ineffective in changing eating and physical activity or makes eating and physical activity worse retrieved no papers. Furthermore, there is a lack of evidence on parameters for BCT effectiveness.

*BCT to change this domain: 4. Social support (emotional)*

To operationalise social support (emotional), a workplace health eating/physical activity support group (with inclusion of family, friends or colleagues) (Leroux, Moore and Dubé, 2013) could be established. An online group discussion forum is a further operationalisation option. To support the use of social support (emotional) for changing eating and physical activity Greaves *et al*. (2011) and the National Institute for Health and Clinical Excellence, (2014) reported evidence of effectiveness. On the other hand, Lyons *et al*. (2014) reported that social support (emotional) was ineffective in changing eating and physical activity. There is a lack of evidence on parameters for social support (emotional) effectiveness.

***Behavioural regulation – TDF identified as important barrier***

*BCT to change this domain: 1. Self-monitoring of behaviour*

Operationalising self-monitoring of behaviour might involve asking nurses to record daily, in a diary, whether they ate healthily/were physically active combined with feedback or providing nurses with a pedometer/ accelerometer and a form for recording daily total number of steps. With regard to effectiveness, several systematic reviews have provided evidence of self-monitoring of behaviour effectiveness in changing eating and physical activity behaviour (Webb *et al*., 2010; Greaves *et al*., 2011; Olander *et al*., 2013; National Institute for Health and Clinical Excellence, 2014; French *et al*., 2014; Lyons *et al*., 2014; Steinberg *et al*., 2015; Teixeira et al., 2015). On the other hand, there is a lack of evidence that self-monitoring of behaviour is ineffective in changing eating and physical activity. However, it should be noted that in a systematic review by French *et al*. (2014) it was found that self-monitoring of behaviour contributed to a worsening of physical activity behaviours. Despite this, the results of French *et al*. (2014) relate to older adults (mean age ≥60 years old), and thus might not be directly relevant to a nursing population. Parameters for effectiveness to highlight and to consider using is self-monitoring of behaviour and subsequent feedback as a combination. Interventions with the combination of self-monitoring of behaviour, goal-setting and action planning have been found to be twice as effective as those that did not (Michie *et al*., 2009).
